# Supplementary figures and images for: Investigating CRISPR/Cas9 gene drive for production of disease-preventing prion gene alleles
Source: PLoS One. 2022 Jun 7;17(6):e0269342. doi: 10.1371/journal.pone.0269342 (PMC9173614; doi:10.1371/journal.pone.0269342)

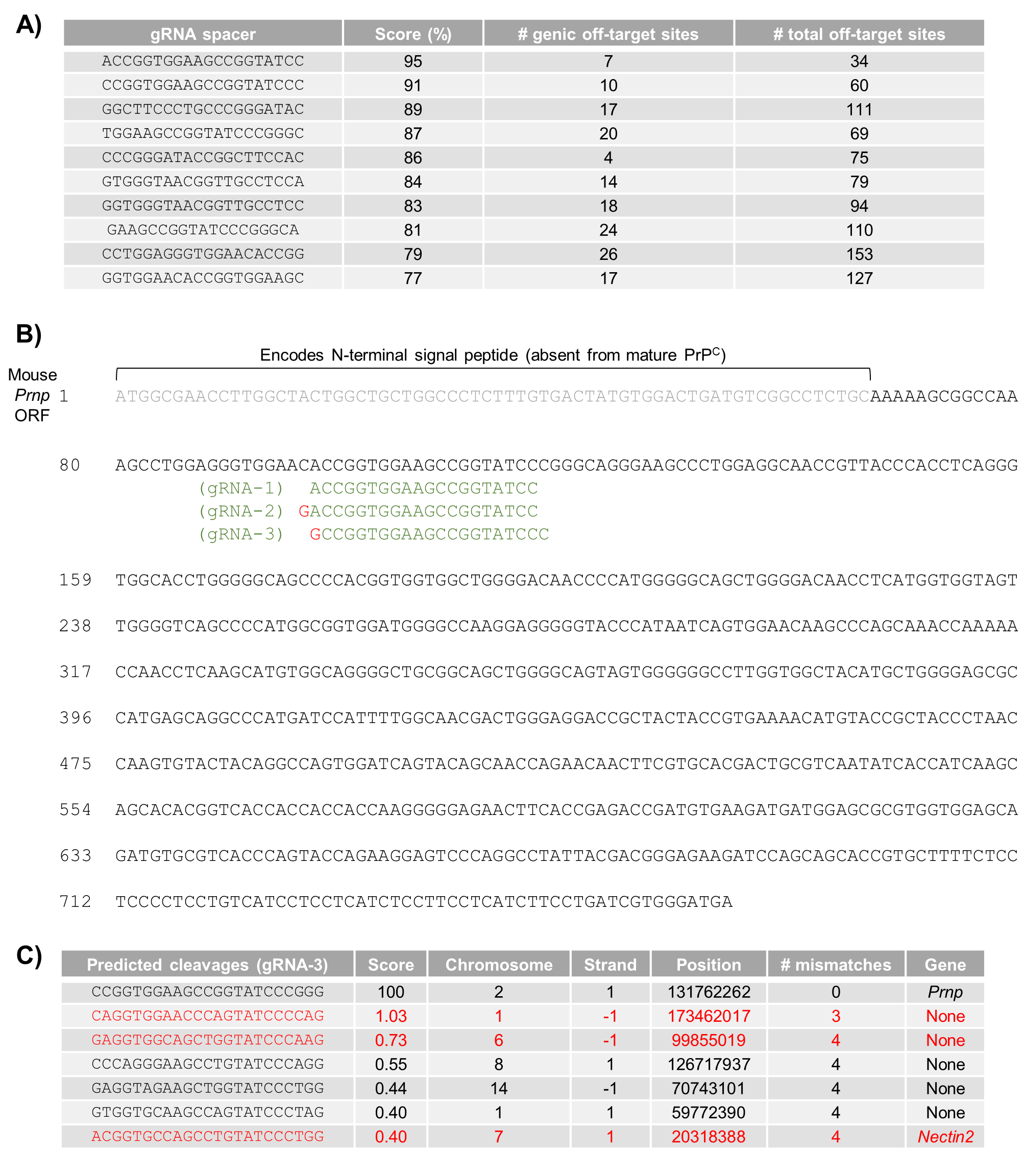

Supplement: S1 Fig — (A) The top 10 gRNA spacers within codons 23–50 of murine Prnp that were identified by the gRNA design algorithm previously found at http://crispr.mit.edu. The scores reported by the algorithm derive from a combination of the on-target efficiency rating and the number of potential off-target sites. (B) The chosen Prnp gRNA spacer sequences are shown aligned to the murine Prnp ORF (NCBI Accession #: NM_011170.3). Due to the preference of the U6 promoter for an initiating G nucleotide, a deliberately mismatched initial G nucleotide was included for gRNA–2 and gRNA–3. (C) The highest-scoring predicted cleavages for the second spacer listed in panel A (contained within gRNA–3). Data derives from the same gRNA design algorithm. Sanger sequencing of a PCR amplicon containing the potential off-target site was attempted for each of the six shown. Red highlighting indicates that suitable primers could be designed and that readable sequencing data was obtained. (TIF) [file pone.0269342.s001.tif]

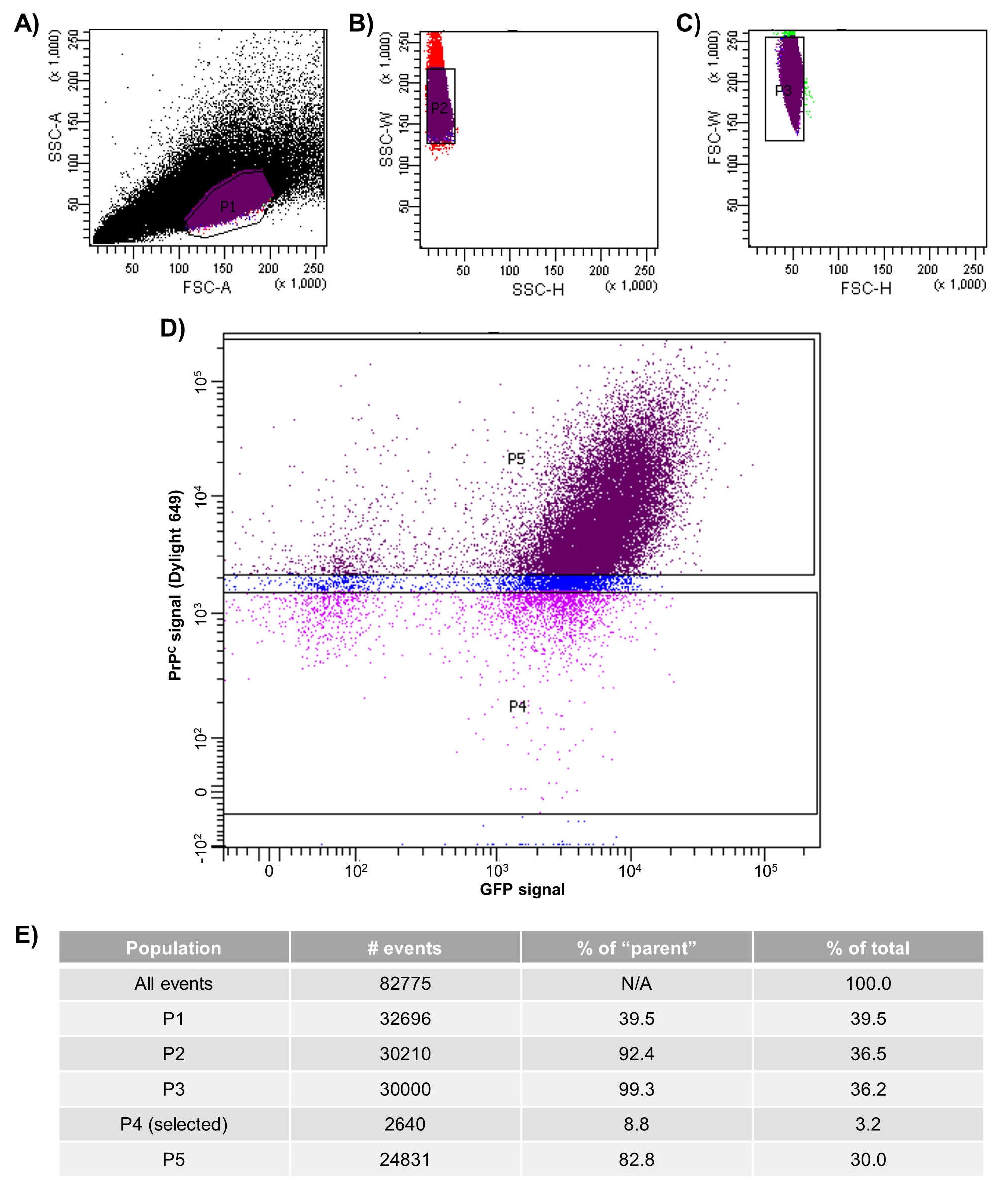

Supplement: S2 Fig — WT-5 RK13 cells underwent FACS 48 h after transfection with eSpCas9(1.1) expression plasmids containing Prnp gRNA–1, –2 or –3, or no gRNA. Example data for gRNA–1 is shown here, but the same process was applied to each sample. Tight gating based on the side scatter area (SSC-A) vs forward scatter area (FSC-A) plot (A) was used to exclude dead cells. (B, C) Gates were applied based on SSC and FSC width (W) vs height (H) plots to exclude cell clumps. (D) A gate was applied to select the ~10% of cells with the lowest PrPC signals (P4). (E) Summary table showing the cell counts in each population. (TIF) [file pone.0269342.s002.tif]

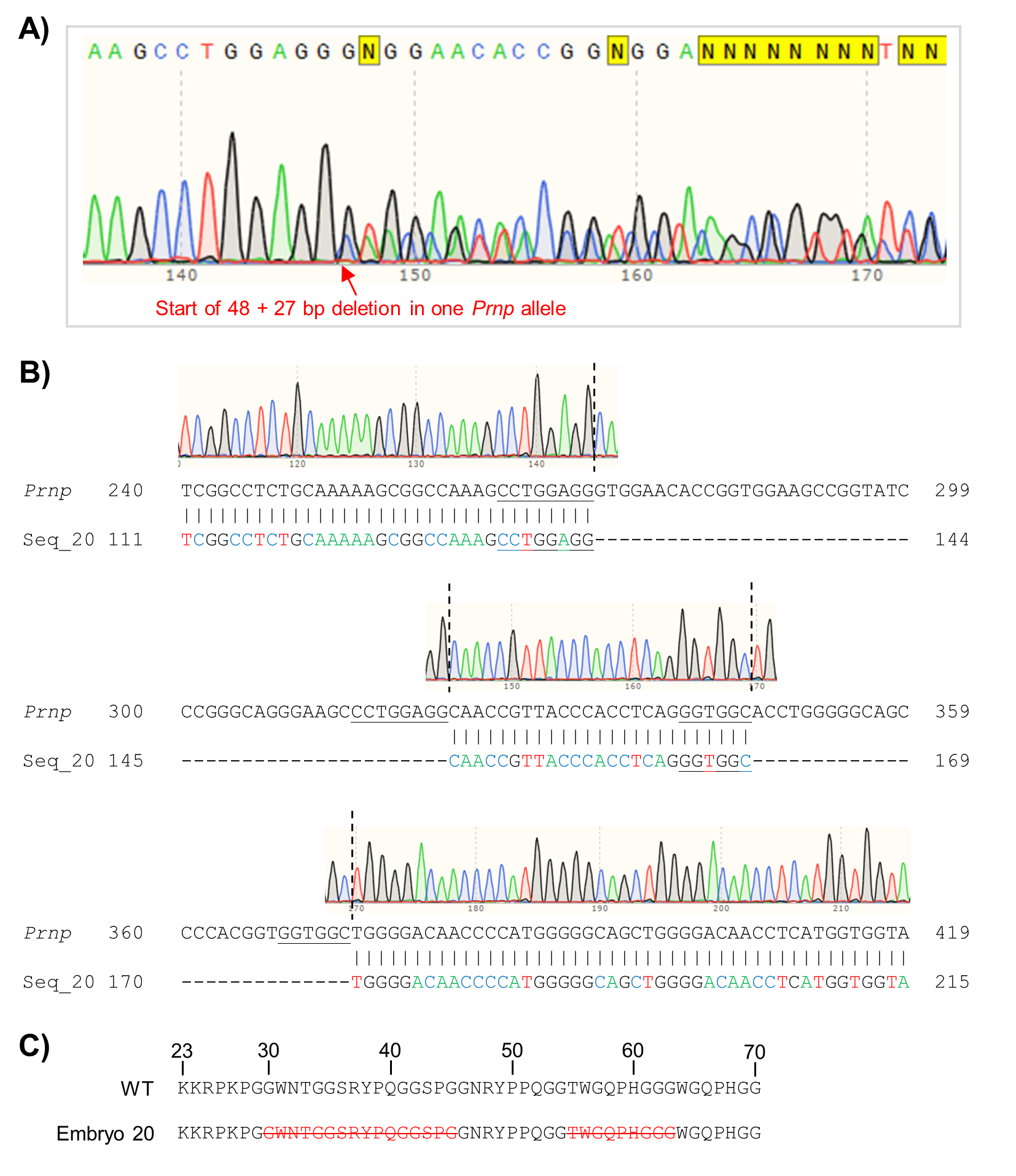

Supplement: S4 Fig — (A) Sanger sequencing chromatogram for embryo 20 derived from fertilized FVB/NJ oocytes that had been electroporated with recCas9/Prnp gRNA–3 RNP complexes. The numbers below the chromatogram indicate the position in the sequencing read rather than the Prnp ORF. The data suggested that two relatively large deletions were present within one Prnp allele. (B) To eliminate the mixed sequence, the original sequencing PCR product was ligated into pCR2.1–TOPO and fresh Sanger sequencing data were obtained from a clone suggested to contain the deletion(s) by diagnostic restriction digests. The alignment with the WT Prnp ORF sequence confirms that two deletions of 48 and 27 bp were present in one Prnp allele of embryo 20. The exact start and end sites of the deletions cannot be determined due to the presence of microhomologies (underlined). (C) The predicted changes to the N-terminal amino acid sequence of mature PrPC in embryo 20 based on the deletions observed within the Prnp ORF. (TIF) [file pone.0269342.s004.tif]
