# Supplementary material for: Investigating CRISPR/Cas9 gene drive for production of disease-preventing prion gene alleles
Source: PLoS One. 2022 Jun 7;17(6):e0269342. doi: 10.1371/journal.pone.0269342 (PMC9173614; doi:10.1371/journal.pone.0269342)
Supplement: S3 Fig — (A) Diagnostic digests of pCR2.1–TOPO containing junction PCR products (purified from the gel shown in Fig 3C) were prepared using the indicated restriction enzymes. The expected band sizes were 4.0 and 1.3 kb or 5.1 and 0.25 kb for the 3’ junction PCR products (depending on the orientation in which the PCR product inserted into pCR2.1–TOPO) and 3.9 and 1.2 kb for the 5’ junction products (irrespective of orientation in pCR2.1–TOPO). All of the 5’ junction samples and 5/6 of the 3’ junction samples produced the expected band patterns. (B, C) Example Sanger sequencing chromatograms for minipreps #1 and #7 showing that the sequences obtained matched the expected junction PCR products, indicating the absence of unwanted indels around the junction sites. For the expected sequences, underlined letters correspond to the Gfp-GPI transgene sequence and non-underlined capitals to the Prnp homology arms. (PDF) [file pone.0269342.s003.pdf]

A)

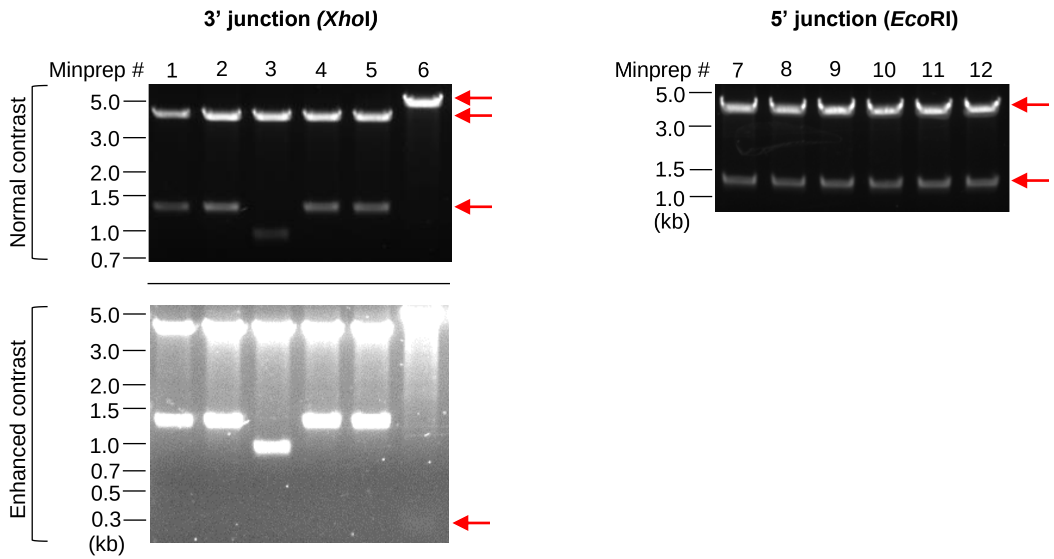

B)

Minprep 1 (3' junction)

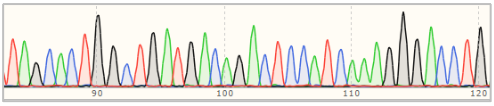

```
Seq_1   61    GATATCTGCAGAATTCGCCCTTTAGCACTGGCTGATGACAGACTCCATCAAAGGGACCTG   120
                                    ||||||||||||||||||||||||||||||||||||||
PCR     1423  ----------------------tagcactggctgatgacagactccatcaaagggACCTG   1386
```

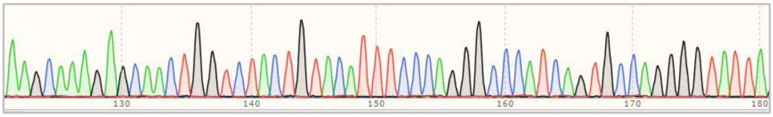

```
Seq_1   121   AAGCAAAGAGCAACTGGTCTACTGTACATTTCCCAGGGCCCATCAGTGCCAGGGGTATTA   180
              ||||||||||||||||||||||||||||||||||||||||||||||||||||||||||||
PCR     1385  AAGCAAAGAGCAACTGGTCTACTGTACATTTCCCAGGGCCCATCAGTGCCAGGGGTATTA   1326
```

Sequenced using M13-For(-20) primer

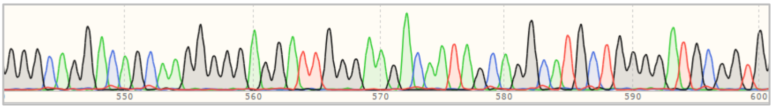

```
Seq_2   541   GGGCAGGACAGCAAGGGGGAGGATTGGGAAGACAATAGCAGGCATGCTGGGGATGCGGTG   600
              ||||||||||||||||||||||||||||||||||||||||||||||||||||||||||||
PCR     478   GGGCAGGACAGCAAGGGGGAGGATTGGGAAGACAATAGCAGGCATGCTGGGGATGCGGTG   537
```

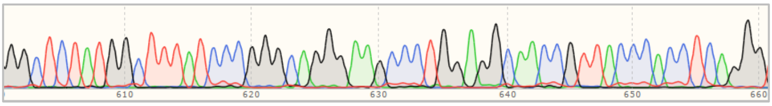

```
Seq_2   601   GGCTCTATGGCTTTATCCCGGGCAGGGAAGCCCTGGAGGCAACCGTTACCCACCTCAGGG   660
              ||||||||||||||||||||||||||||||||||||||||||||||||||||||||||||
PCR     538   GGCTCTATGGCTTTATCCCGGGCAGGGAAGCCCTGGAGGCAACCGTTACCCACCTCAGGG   597
```

Sequenced using M13-Rev primer

C)

Minprep 7 (5’ junction)

Sequenced using M13-For(-20) primer

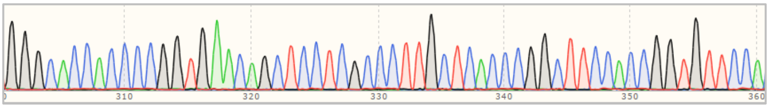


```
Seq_1   301   GGGCACCACCCCGGTGAACAGCTCCTCGCCCTTGCTCACCCGGCTTCCACCGGTGTTCCA   360
              ||||||||||||||||||||||||||||||||||||||||||||||||||||||||||||
PCR     976   GGGCACCACCCCGGTGAACAGCTCCTCGCCCTTGCTCACCCGGCTTCCACCGGTGTTCCA   917
```

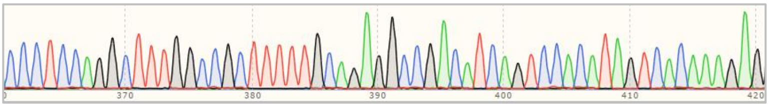


```
Seq_1   361   CCCTCCAGGCTTTGGCCGCTTTTTGCAGAGGCCGACATCAGTCCACATAGTCACAAAGAG   420
              ||||||||||||||||||||||||||||||||||||||||||||||||||||||||||||
PCR     916   CCCTCCAGGCTTTGGCCGCTTTTTGCAGAGGCCGACATCAGTCCACATAGTCACAAAGAG   857
```

Sequenced using M13-Rev primer

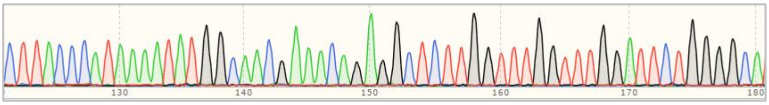


```
Seq_2   121   CTTACCCATAAAATATGGCAACGAAACAGAGGCTCTTGGTTTGGTTTGGATTCTGGGGCA   180
              ||||||||||||||||||||||||||||||||||||||||||||||||||||||||||||
PCR     60    cttacccataaaatatggcaacgaaacagaggctcttggtttggtttggattctggggca   119
```

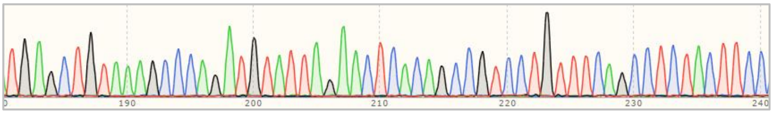


```
Seq_2   181   TGAGCTGTAAAGCCCAGATGTATTAGAACTCACAGCCGTCCTGTTTCAGCCTCTACTTCC   240
              ||||||||||||||||||||||||||||||||||||||||||||||||||||||||||||
PCR     120   tgagctgtaaagcccagatgtattagaACTCACAGCCGTCCTGTTTCAGCCTCTACTTCC   179
```
