# Supplementary material for: Investigating CRISPR/Cas9 gene drive for production of disease-preventing prion gene alleles
Source: PLoS One. 2022 Jun 7;17(6):e0269342. doi: 10.1371/journal.pone.0269342 (PMC9173614; doi:10.1371/journal.pone.0269342)
Supplement: S5 Fig — The figure panels that the images correspond to are indicated. A red “X” is used to indicate that a lane was omitted from the final figure panel. (PDF) [file pone.0269342.s005.pdf]

Agarose gel 1

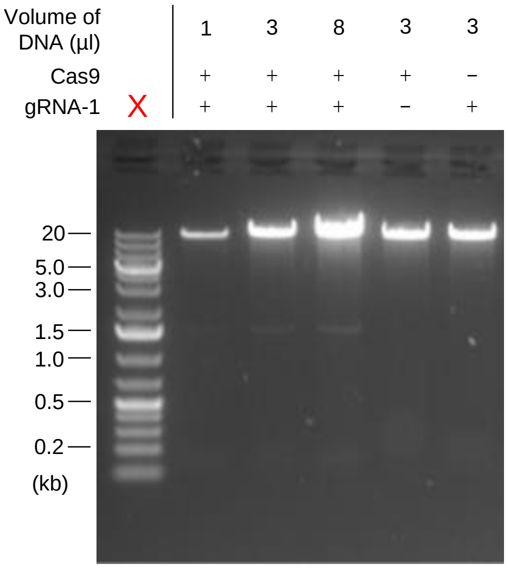

Captured using a Fluor Chem E Imager (ProteinSimple). Used to generate Fig. 2A.

Agarose gel 2

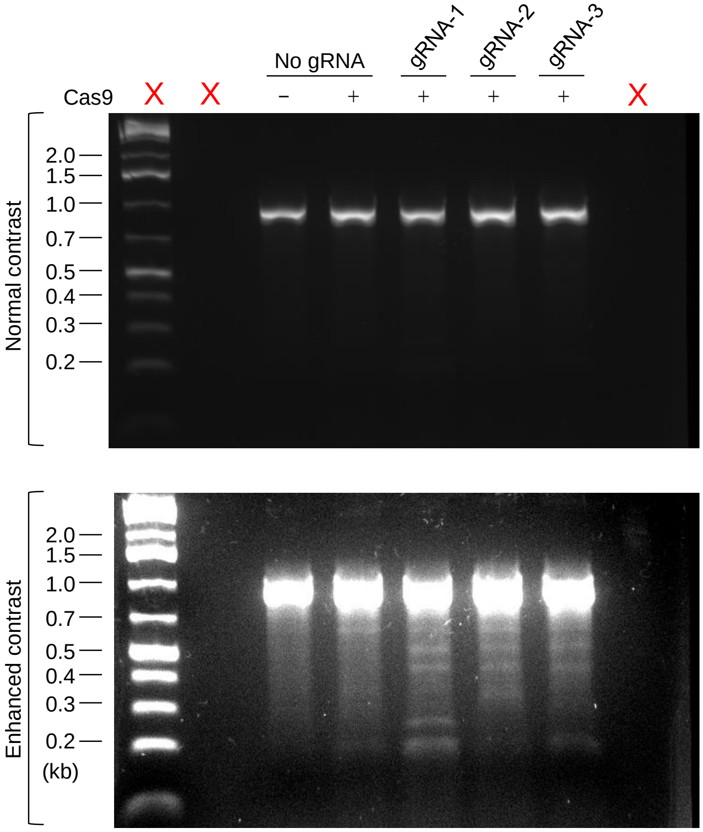

Captured using a Fluor Chem E Imager (ProteinSimple). Used to generate Fig. 2B.

Agarose gel 3

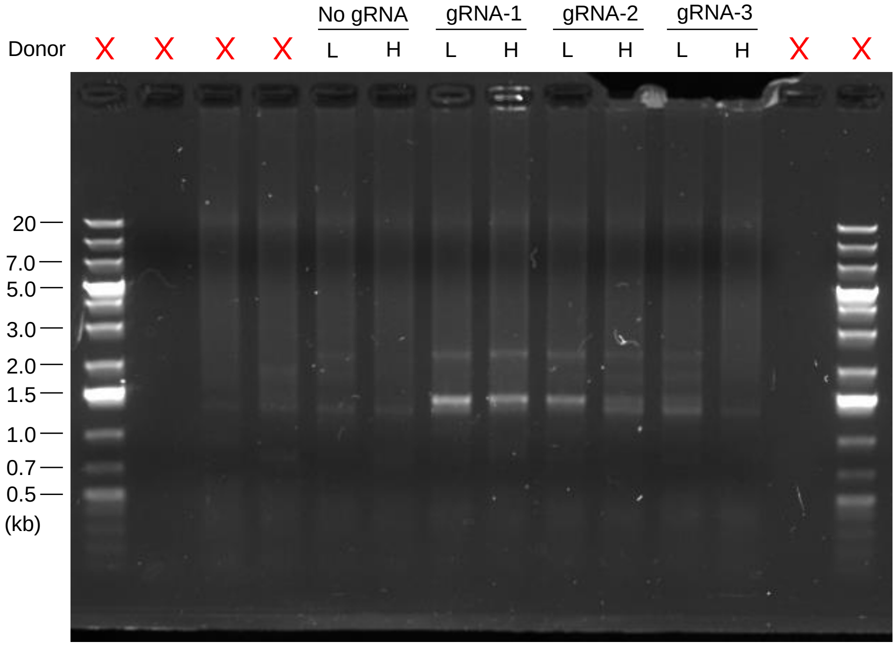

Captured using a Fluor Chem E Imager (ProteinSimple). Used to generate Fig. 3B.

Agarose gel 4

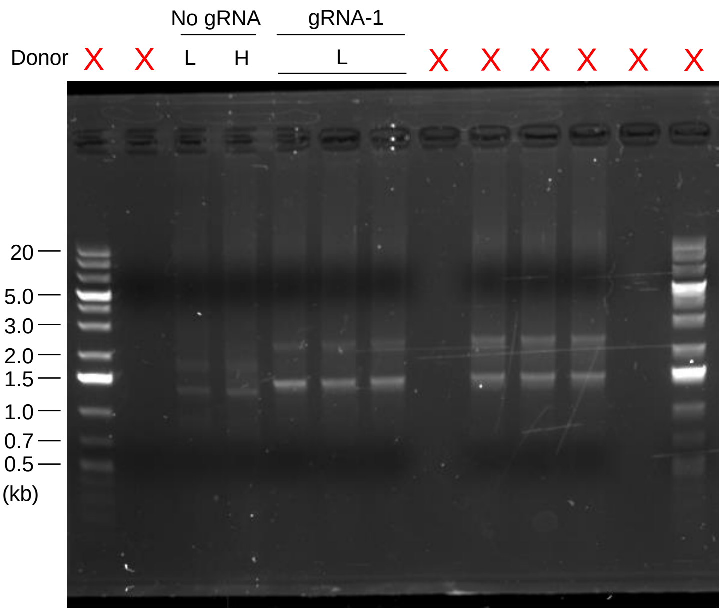

Captured using a Fluor Chem E Imager (ProteinSimple). Used to generate the top half of Fig. 3C.

Agarose gel 5

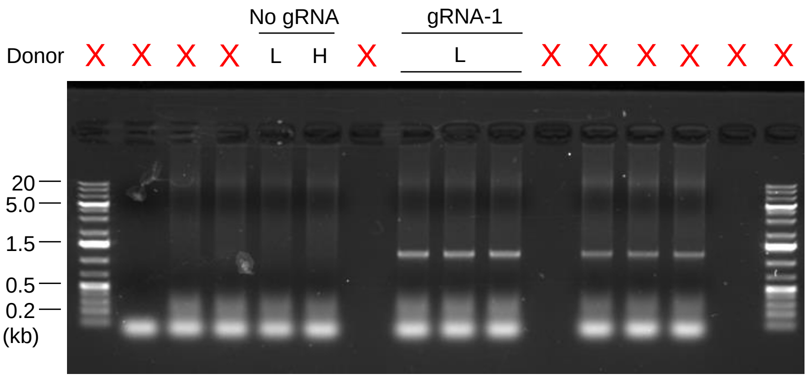

Captured using a Fluor Chem E Imager (ProteinSimple). Used to generate the bottom half of Fig. 3C.

Agarose gel 6

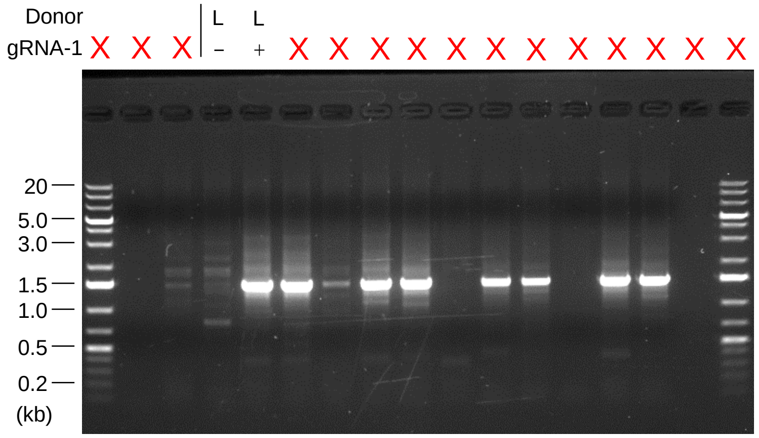

Captured using a Fluor Chem E Imager (ProteinSimple). Used to generate the top half of Fig. 3D.

Agarose gel 7

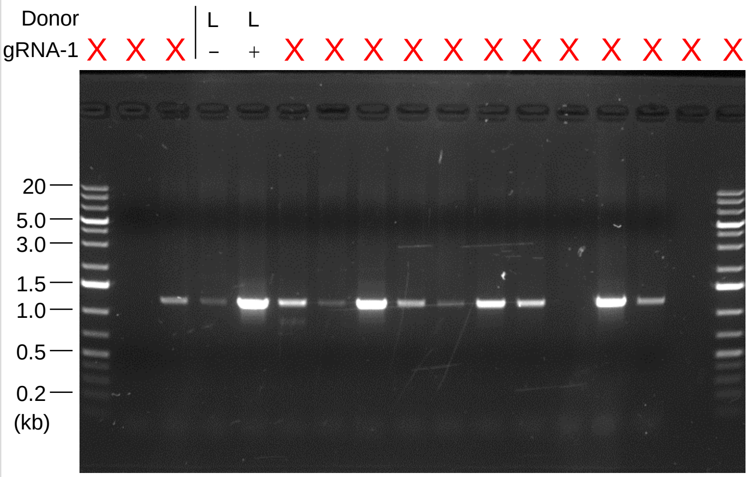

Captured using a Fluor Chem E Imager (ProteinSimple). Used to generate the bottom half of Fig. 3D.

Agarose gel 8

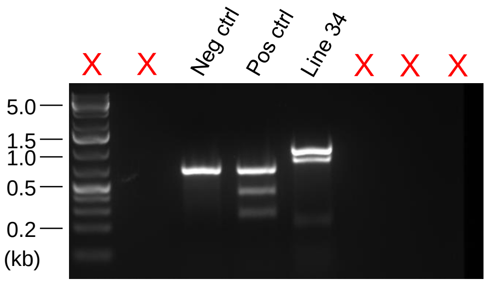

Captured using a Fluor Chem E Imager (ProteinSimple). Used to generate Fig. 4B.

Agarose gel 9

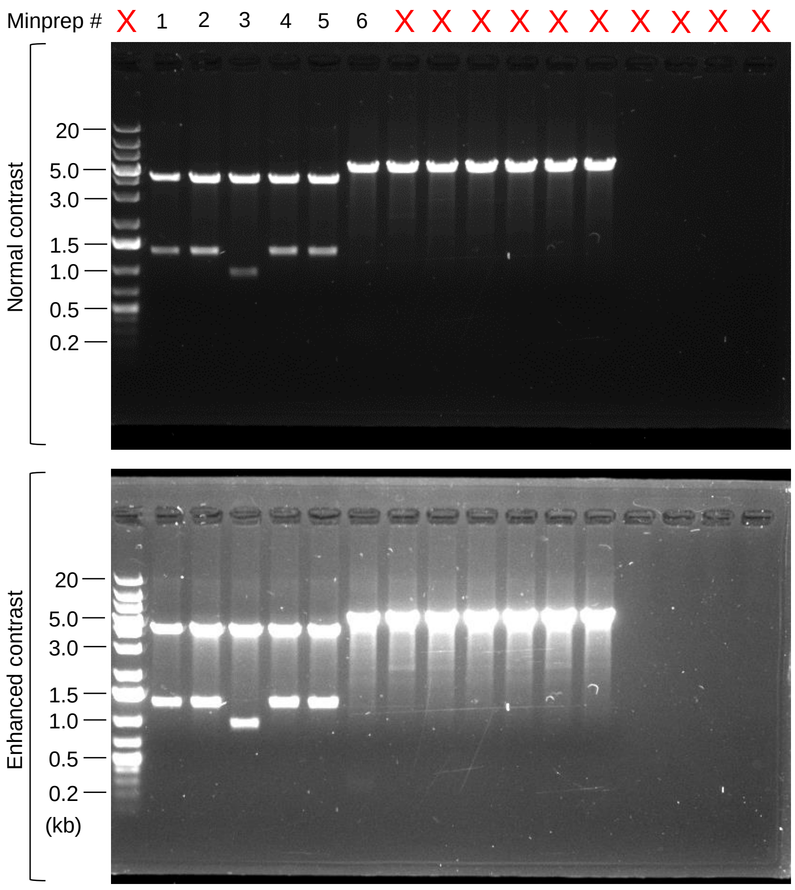

Captured using a Fluor Chem E Imager (ProteinSimple). Used to generate the left hand side of Fig. S3A.

Agarose gel 10

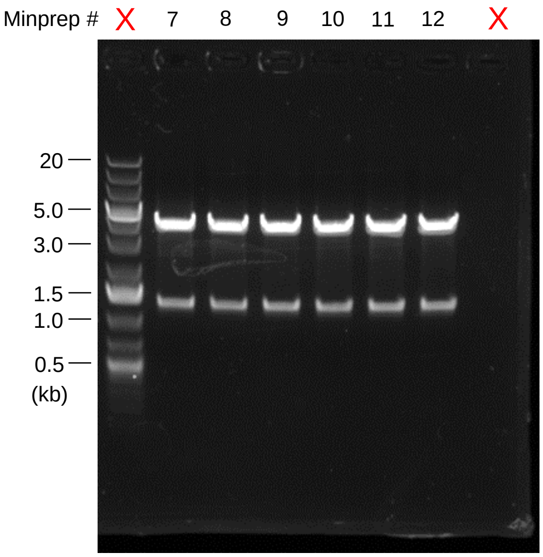

Captured using a Fluor Chem E Imager (ProteinSimple). Used to generate the right hand side of Fig. S3A.

Capillary western image 1

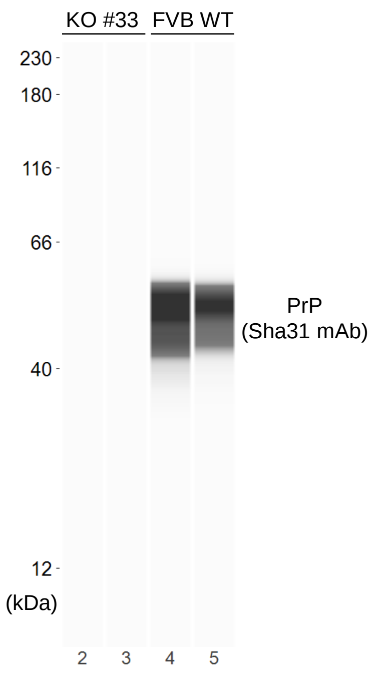

Captured using a Wes instrument (ProteinSimple). Used to generate the top part of Fig. 4C.

Capillary western image 2

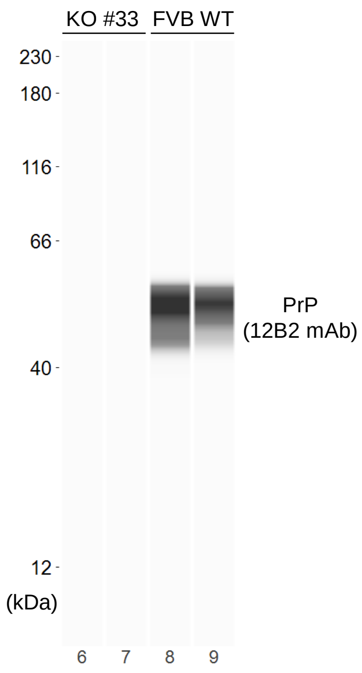

Captured using a Wes instrument (ProteinSimple). Used to generate the middle part of Fig. 4C.

Capillary western image 3

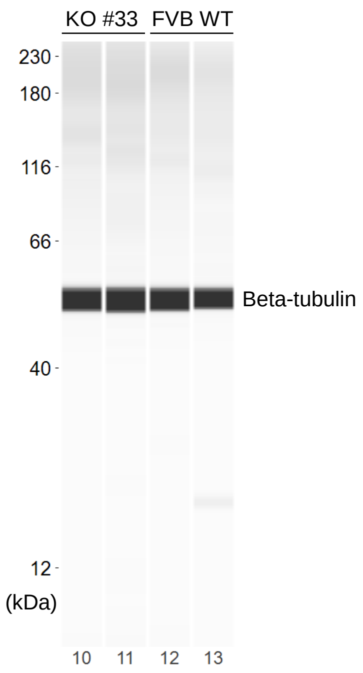

Captured using a Wes instrument (ProteinSimple). Used to generate the bottom part of Fig. 4C.

Capillary western image 4

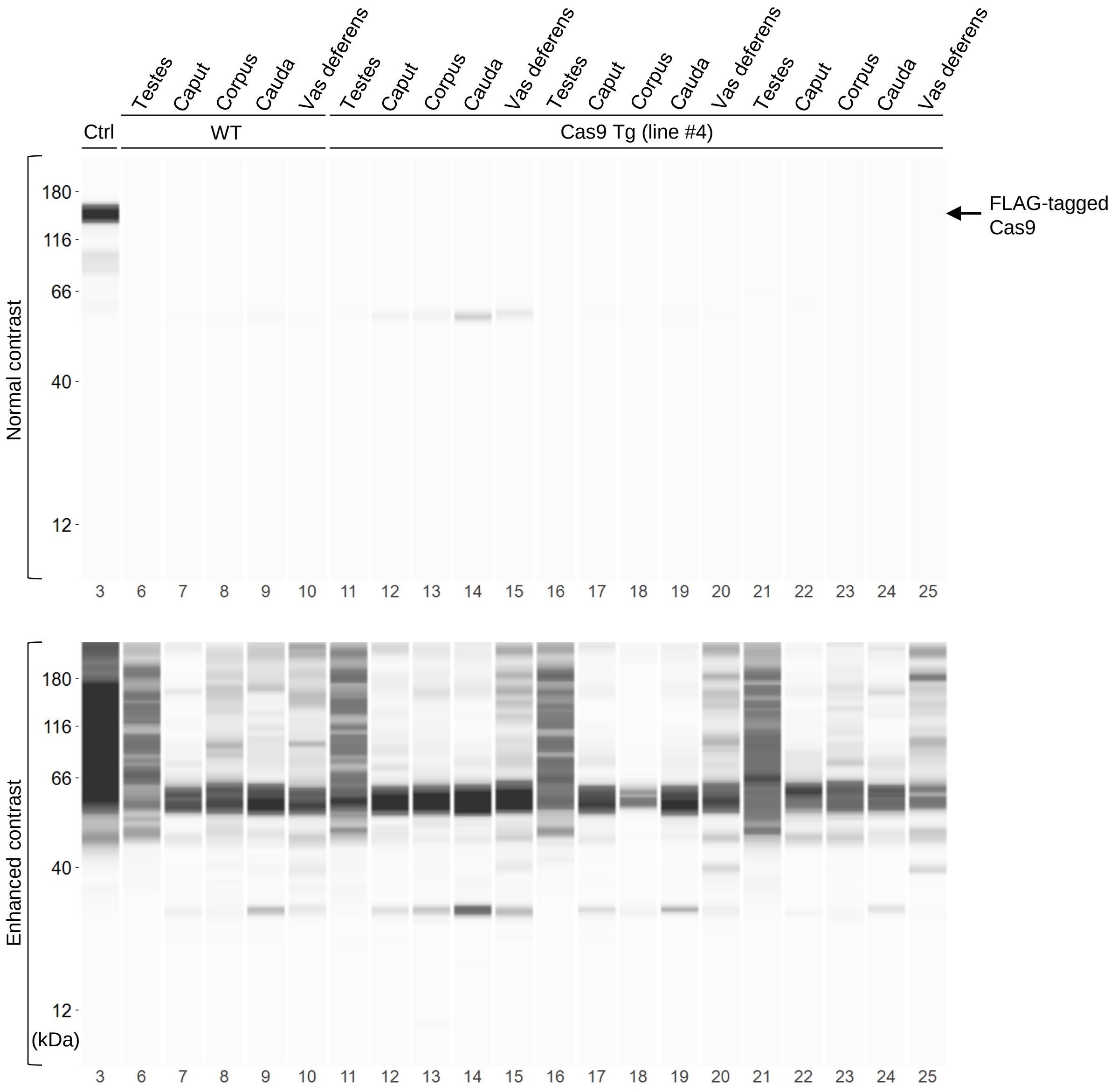

Captured using a Wes instrument (ProteinSimple). Used to generate Fig. 5A.

Capillary western image 5

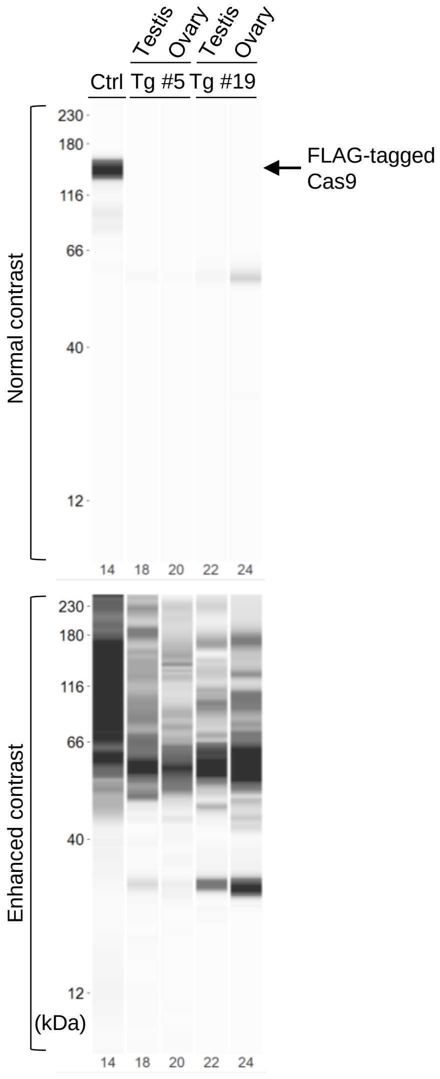

Captured using a Wes instrument (ProteinSimple). Used to generate the top half of Fig. 5B.

Capillary western image 6

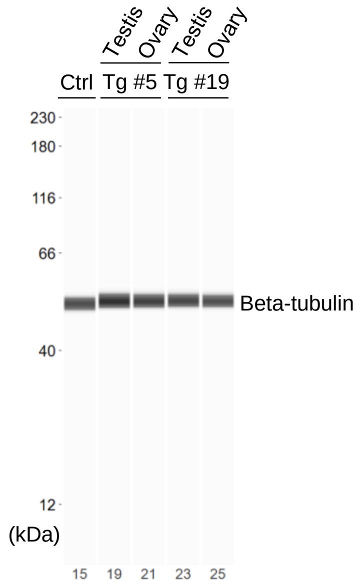

Captured using a Wes instrument (ProteinSimple). Used to generate the bottom half of Fig. 5B.

Capillary western image 7

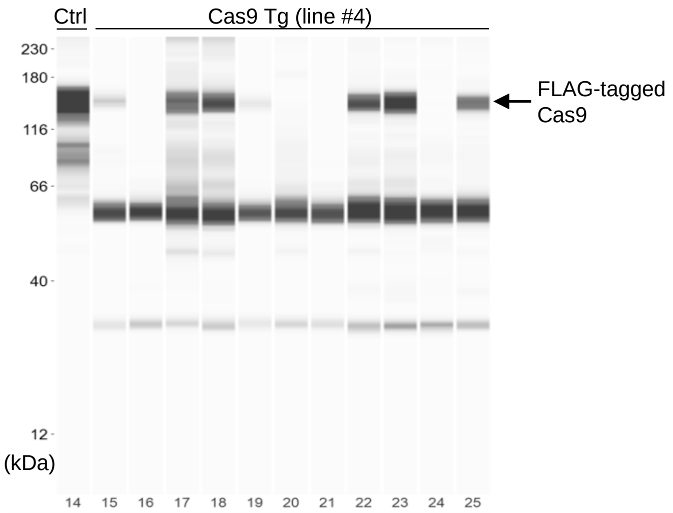

Captured using a Wes instrument (ProteinSimple). Used to generate the top half of Fig. 5C.

Capillary western image 8

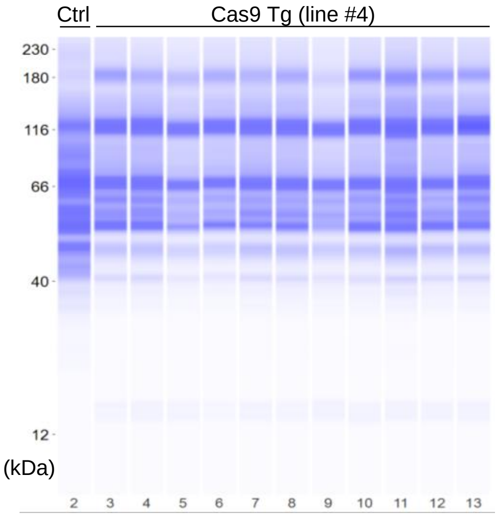

Captured using a Wes instrument (ProteinSimple). Used to generate the bottom half of Fig. 5C.
