# Supplementary material for: Investigating CRISPR/Cas9 gene drive for production of disease-preventing prion gene alleles
Source: PLoS One. 2022 Jun 7;17(6):e0269342. doi: 10.1371/journal.pone.0269342 (PMC9173614; doi:10.1371/journal.pone.0269342)
Supplement: S1 Table — The ends of the gBlock fragment (underlined) were complementary to the pBud.GFP forward (For) and reverse (Rev) primers to facilitate Gibson Assembly; the sequence in lower case corresponds to codons 230–254 of murine Prnp. The PrnpHA–For and–Rev primers both start with 6 random nucleotides followed by a SalI site (For) or HindIII site (Rev), the protospacer sequence plus the protospacer-adjacent motif for Prnp gRNA–3 (see S1 Fig, panel B, for sequence), and, finally, the Prnp-specific sequences. To facilitate Gibson Assembly, the GFP–GPI–For1 and–Rev1 primers contained 18 nt 5’ sequences complementary to the ends of the vector fragment amplified by the pB–HA–For and–Rev primers. For the junction PCRs, the Prnp–Intron2–For and GFP–GPI–Rev2 primers were used to analyse the 5’ junction, GFP–GPI–For2 and Prnp–3UTR–Rev the 3’ junction. The M13–For(-20) and M13–Rev primers were provided as part of a TOPO TA cloning kit (Invitrogen, 450641) and were used to sequence the junction PCR products in both directions. (PDF) [file pone.0269342.s006.pdf]

S1 Table. Primer and DNA sequences

**gBlock Fragment**	**Sequence**
Prnp230–254 (stage 1)	GGCATGGACGAGCTGTACAAGtccagcagcaccgtgcttttctcctcccctcctgtcatcctcctcatctccttcctcatcttcctgatcgtgggaTAAAGCGGCCGCTTCGAAGGTACCAGCACAGTGGACTCGAGAGAT
**Primer (cloning stage)**	**Sequence**
For generating donor vector for HDR	
pBud.GFP–For (stage 1)	AGCACAGTGGACTCGAGAGAT
pBud.GFP–Rev (stage 1)	CTTGTACAGCTCGTCCATGC
PrnpHA–For (stage 2)	ACATAT GTCGAC CCGGTGGAAGCCGGTATCCCGGG AACTCACAGCCGTCCTGTTTCAGC
PrnpHA–Rev (stage 2)	ACAGTT AAGCTT CCGGTGGAAGCCGGTATCCCGGG ACCTGAAGCAAAGAGCAACTGGTC
GFP–GPI–For1 (stage 3)	AACACCGGTGGAAGCCGG GTGAGCAAGGGCGAGGAGCTG
GFP–GPI–Rev1 (stage 3)	GCTTCCCTGCCCGGGATA AAGCCATAGAGCCCACCGCATC
pB–HA–For (stage 3)	TATCCCGGGCAGGGAAGCC
pB–HA–Rev (stage 3)	CCGGCTTCCACCGGTGTTC
For generating Prl3b1-Cas9 expression construct	
*Xma*I–bGHpA–For (stage 1)	CCCGGGCGACTGTGCCTTCTAGTTGC
*Spe*I–bGHpA–Rev (stage 1)	ACTAGTCCATAGAGCCCACCGCATCC
*Hind*III–Intron–For (stage 2)	AAGCTTGGATCCTGAGAACTTCAGGG
*Pst*I–Intron–Rev (stage 2)	CTGCAGTTTGCCAAAATGATGAGACA
*Kpn*I–*Not*I–LCR–For (stage 3)	GGTACCGCGGCCGCAAGCTTCTGACAAATTATTC
*Xho*I–LCR–Rev (stage 3)	CTCGAGGGATCCTCCCATTTCGGCCT
Linker–For (5’-phos.) (stage 4)	TCGAGAAGGCGCGCCAACATATGAAGACGTCAAGCTAGCAAAT
Linker–Rev (5’ phos.) (stage 4)	CGATTTGCTAGCTTGACGTCTTCATATGTTGGCGCGCCTTC
*Age*I–Linker–1 (5 phos.) (stage 5)	GACCGGTC
*Age*I–Linker–2 (5 phos.) (stage 5)	CCGGAACCGGTCTGCA
*Xho*I–Prl3b1p–For (stage 7)	AAACTCGAGGAAAAAGTTGAAATAATATCAAAATC
*Nhe*I–Prl3b1p–Rev (stage 7)	AAAGCTAGCCCCCGAGTGCTGCCTTCC
For WT-5 RK13 T7E1 assays	
RK13–T7E1–For	CATCAATGGGCGTGGATAGC
RK13–T7E1–Rev	CCATCATCTTCACATCGGTCTC
For amplification and sequencing of Prnp in mice	
Mo–T7E1–For	ACATTTGCTTTGTAGATACATGTCA
Mo–T7E1–Rev	CCATCAGTGCCAGGGGTATT
For junction PCRs	
Prnp–Intron2–For	GGATGTTAATTCCGTCACTTGAC
GFP–GPI–Rev2	ACTTGAAGAAGTCGTGCTGCTTC
GFP–GPI–For2	AACGAGAAGCGCGATCACATG
Prnp–3UTR–Rev	TAGCACTGGCTGATGACAGAC
For sequencing of junction PCR products	
M13–For(-20)	GTAAAACGACGGCCAG
M13–Rev	CAGGAAACAGCTATGAC
