# Supplementary material for: Investigating CRISPR/Cas9 gene drive for production of disease-preventing prion gene alleles
Source: PLoS One. 2022 Jun 7;17(6):e0269342. doi: 10.1371/journal.pone.0269342 (PMC9173614; doi:10.1371/journal.pone.0269342)
Supplement: Attachment — Submitted filename: Response to Reviewers_PLOS ONE.docx [file pone.0269342.s007.docx]

Centre for Prions and Protein Folding Diseases,

2-04 Brain and Aging Research Building,

University of Alberta,

Edmonton, Alberta T6G 2M8

Phone:(780)-492-9024

Email david.westaway@ualberta.ca

May 03, 2022

Dear Dr. Morales,

We thank you and the reviewers for the assessment of our manuscript entitled:

“Investigating CRISPR/Cas9 gene drive for production of disease-preventing prion gene alleles”

Based on the comments received, we have made a number of adjustments to the manuscript. Detailed changes are outlined below:

**Responses to comments from reviewer #1**

1. “Figure 2. The numbering of the lines is not present in the figure and gRNA nomenclature is not identical in the figure (#1, #2, #3) and in the figure legend (-1, -2, -3)”. The nomenclature in Figure 2 and its legend has been made consistent (similar changes have been made to Figure 3). We assume that “lines” is a typo and that the comment refers to the lane numbering originally provided in the Figure 2 legend; references to lane numbers have been removed from the legend to avoid confusion.
2. “Figure S1 (B). Define clearly the spacer sequence (#1) used in this work”. In fact, all three spacer sequences are used at some point in the study. #3 was used for the in vivo work, but all three were used for the experiments in the RK13 and N2a cells (Figures 2 and 3).

**Responses to comments from reviewer #2**

1. The authors designed the gRNAs based on mouse *Prnp* sequence, and tested their efficiency and specificity in mouse N2a cells expressing wild type mouse PrP, and rabbit RK13 cells and mule deer brain MDB cells both engineered to express mouse PrP gene. Could the authors clarify if the gRNAs also target rabbit and mule deer *Prnp*? There are several mismatches between the rabbit *Prnp* DNA sequence and the gRNA spacer sequences used here, so cleavage of the rabbit *Prnp* by Cas9 would be unlikely. Also, just in case there was any confusion, the T7E1 assays performed on genomic material from the RK13 cells (reported in Figure 2B) were designed solely to pick up disruptions to mouse *Prnp*, as the forward primer for PCR amplification targets a sequence present in the vector backbone of the stably integrated mouse *Prnp* expression construct. Some minor edits have been made to the second results section to clarify that we were targeting the mouse *Prnp* in these cells.

As for the MDB cells, these were not actually engineered to express mouse *Prnp*; instead, we were targeting the endogenous cervid *Prnp* and different gRNAs were designed for this purpose. We discovered through use of transfection control plasmids (e.g., GFP) that the issue with these experiments was the very low transfection efficiency. Therefore, we do not think there is any value in reporting the gRNA spacer sequences used (because we do not know whether they work or not).

2. In both RK13 and MDB cells, the experiments did not achieve satisfactory results, for MDB cells, it was because the low transfection efficiency; in RK13 cells, the reason for the suboptimal results was due to low levels of Cas9 cleavage. Could the authors discuss what strategies could be used in the future to improve the research? We would argue that the results in the RK13 cells were sufficient in that they enabled us to move onto the next step (i.e., assessing activity in vivo), where the gRNAs were shown to work quite effectively. However, we have added some comments to the discussion section about potential improvements to these experiments as well as the experiments assessing cleavage of cervid *Prnp* (lines 264–274).
3. It is known that some PrP gene mutations could lead to spontaneous prion disease. How can the researchers achieve complete elimination of PrP gene expression without causing pathological gene alterations of PrP in animals? It is important to point out that if the gene drive system worked perfectly then PrP expression should be eliminated entirely rather than a mutant form being expressed. However, it is true that no drive can have perfect efficiency and that sometimes the DNA will be repaired by error-prone mechanisms, leading to mutations. Although certain single amino acid changes are associated with the development of spontaneous prion disease in humans, these tend to be far away from the expected Cas9 cleavage site for our chosen gRNAs (most are within the globular C-terminal domain). However, as we discuss in the second half of the Discussion section, we did discover that use of our gRNAs sometimes causes in-frame deletions resulting from microhomology-mediated end-joining repair (due to the repetitive nature of much of the PrP N-terminal domain). Deletions including part of the PrP N-terminal can cause lethal neurodegenerative phenotypes, although these do not result from a prion disease, and, in any case, the deletion must stretch all the way into the hydrophobic core of the protein (residues ~112–133) to cause these phenotypes; we consider it unlikely that repair of a Cas9-generated double-strand break could cause such a long deletion, although it is possible. We have added some discussion on this point to the manuscript (lines 340–344). This section cites two new references: Shmerling et al. (1998) and Baumann et al. (2007).

Certainly, if a gene drive system targeting *Prnp* could be made to work effectively in mammals, pushing the efficiency as high as possible would be critical (to reduce the chance of a pathological mutation occurring). Also, it is important to point out that repair by classical NHEJ or microhomology-mediated end-joining does not rely on a separate homologous template; hence, any mutant alleles generated should not have the gene drive functionality and so would not spread among the population in a super-Mendelian fashion.

4. Could the authors please update if these gene mutant mouse lines (Lines 33, 34 and 36) could develop normally? Are there any abnormal phenotypes during aging? Although detailed characterization of these lines was not performed, the mice appeared phenotypically normal and no breeding abnormalities were noted. A sentence attesting to this fact has been added (lines 193–194). We have not aged the mice beyond a few months of age, but we would expect Line 33 (which carries the frameshift mutation) to develop the demyelinating polyneuropathy that has been observed from middle-age onwards in several lines of PrP^C^-null mice, including the coisogenic ZH3 line recently generated using TALEN gene editing technology (Nuvolone et al. 2016, JEM).

**Other points relating to journal requirements**

- Minor formatting changes have been made to the manuscript file to match the PLOS style requirements.
- Figures were checked using the recommended PACE tool.
- Original blot and gel images are presented as Supporting Information (S5 Fig).

We hope that with these adjustments our paper is now suitable for publication.

Sincerely,

David Westaway PhD FCAHS

Director, Centre for Prions and Protein Folding Diseases,

Canada Research Chair (Tier1) Prion Biology.

Professor (Neurology), Department of Medicine, University of Alberta
